
JOURNAL INFORMATION
==============================
NLM Title Abbreviation: Function (Oxf)
Journal ID: Function (Oxf)
NLM Journal ID: 101770668
Journal ID: function

Function (Oxford, England)

EISSN: 2633-8823

ARTICLE INFORMATION
==============================
PMCID: PMC7543493
PMID: 35321210
DOI: 10.1093/function/zqaa019
Article ID: zqaa019
Article version: 1
Subjects: Opinion Article

Thinking through FUNCTION during COVID-19

Molina Patricia E pmolin@lsuhsc.edu
Department of Physiology, School of Medicine, Louisiana State University Health Sciences Center – New Orleans
Corresponding author. 1901 Perdido Street, Room 7205, Box P7-3, New Orleans, LA 70112-1393, USA. Email: pmolin@lsuhsc.edu
Electronic publication date: 2020 Sep 26
Electronic Location ID: zqaa019
Received 2020 Sep 11; Revised 2020 Sep 14; Accepted 2020 Sep 14
Copyright: © The Author(s) (2020). Published by Oxford University Press on behalf of American Physiological Society.
Copyright year: 2020
License: This is an Open Access article distributed under the terms of the Creative Commons Attribution License (http://creativecommons.org/licenses/by/4.0/), which permits unrestricted reuse, distribution, and reproduction in any medium, provided the original work is properly cited.
License URL: https://creativecommons.org/licenses/by/4.0/

Page count: 6
edited-state: accepted-manuscript
article-lifecycle: PAP

==============================
FUNCTION™
american physiological society

### Thinking through FUNCTION during COVID-19

Patricia E. Molina [1*]

Department of Physiology, School of Medicine, Louisiana State University Health Sciences Center – New Orleans

*Corresponding author. 1901 Perdido Street, Room 7205, Box P7-3, New Orleans, LA 70112-1393, USA. Email: pmolin@lsuhsc.edu

As the summer of 2020 comes to an end, we find ourselves amid uncharted waters, facing a drastic shift in our teaching, mentoring, and research strategies. The coronavirus disease 2019 (COVID-19) pandemic has challenged administrators, health care providers, scientists, and the lay community at large. Much attention has been devoted to SARS-CoV-2, its trajectory, its potential therapeutic strategies, and the role of masks and social distancing in containing the pandemic. For researchers and health care providers, the quest for understanding the diversity in symptomatology presentation and pathology associated with infection and disease progression is an acute reminder of the centrality that organismal and cellular FUNCTION play in elucidating mechanisms that may be targeted in management of disease. In addition, incrementally we are appreciating that the multiformity of clinical manifestations — ranging from no symptoms to death — can be affected by genetics, environment, lifestyle, and pre-existing conditions. In my opinion, as scientists amid so much uncertainty, our thinking and critical observations of facts coupled with our fundamental knowledge lends itself to consider novel areas of research prime for discovery.

Race and ethnicity have been identified as increased risk factors for COVID mortality, with African American and Hispanics having higher mortality than Caucasian Americans. In addition, health disparities resulting from racial inequities and structural barriers to health care have been painfully exposed during the pandemic. The underlying biological conditions, coupled with environmental and behavioral factors including unhealthy alcohol use, smoking, and consumption of unhealthy diets, make for the perfect storm (FIG. 1). A biological condition that repeatedly surfaces as a risk factor for COVID-19-related mortality within these populations is obesity.[1] The Centers for Disease Control and Prevention (CDC), now lists obesity among the high-risk medical conditions for developing severe illness from COVID-19, ranked among immunocompromised state, chronic kidney disease, and COPD.[2] The link between obesity and significantly high risk of mortality from COVID-19 infection has naturally become a major source of questioning for researchers.

Scientific evidence shows that obesity is characterized by chronic low-level inflammation, a trait that has been associated with insulin resistance and metabolic dysregulation. More recently, evidence suggests that metabolic dyshomeostasis may extend beyond the canonical tissues involved in maintaining glucose homeostasis like skeletal muscle, adipose tissue, and the liver to the immune system. This renewed recognition of the importance of energy metabolism of cells of the immune system, or immunometabolism, could be an area of much needed investigation to understand the possible link between metabolic dysregulation, host immune response to the virus, and differential disease severity.

Immunometabolism reflecting the shifts in metabolic pathways that occur upon immune cell activation is a dynamic and adaptive response to infections. The energetics involved in mounting a robust and appropriate inflammatory response to an antigen are significant. The proliferation and activation of immune cells, and their migration and effector functions, are all dependent on a delicate balance between oxidative and glycolytic metabolism.[3] Preferential substrate utilization and metabolic pathway can modulate immune responses. Enhanced glycolysis in activated immune cells supports the increased energy demands required for effector functions like

© The Author(s) 2020. Published by Oxford University Press on behalf of the American Physiological Society.

This is an Open Access article distributed under the terms of the Creative Commons Attribution License (http://creativecommons.org/licenses/by/4.0/), which permits unrestricted reuse, distribution, and reproduction in any medium, provided the original work is properly cited.

phagocytosis, cytokine production, and antigen presentation. In addition, fatty acid synthesis promotes macrophage inflammatory responses while fatty acid oxidation may reduce their inflammatory potential. The increased metabolic demands coupled with the effects of viral proteins on the integrity of the cellular machinery responsible for maintaining metabolic homeostasis can synergize to promote immune cell activation, senescence, and death, crippling the integrity of the host immune system. The extent to which deranged immunometabolism contributes to the underlying systemic pathophysiological processes that lead to multiorgan failure in COVID-related mortality is unknown.

The lung is a primary target for SARS2 infection. However, damage to other organs and processes, including the kidney and inflammatory and coagulation pathways, strongly suggests the possibility of shared explanatory cellular pathophysiological mechanisms. Cellular energetics could fit that bill. The adaptability of immune cell metabolic pathway and substrate preference, or metabolic plasticity, could be particularly susceptible to SARS2 virulence. Differences in flexibility of metabolic adaptation[4] or metabolic plasticity during the period of acute infection could explain differential outcomes among infected individuals. Furthermore, it could provide insight into the underlying factors that determine viral clearance. Reports in the literature support an association between viral load and rate of clearance with severity of disease, where mild cases have an early viral clearance and severe cases remain positive for longer windows of time of onset.[5] A possible mechanism for protracted viral loads and inefficient clearance could be mediated by disrupted immunometabolism.

Supporting evidence comes from reports in the literature showing the ability of viruses to affect mitochondrial homeostatic mechanisms, including metabolism, and consequently enhance conditions that favor viral replication.[6] For example, human cytomegalovirus enhances glycolytic flux, while herpes simplex virus 1 leads to induction of the tricarboxylic acid cycle. It is unclear what effects coronavirus exerts on mitochondrial homeostasis or metabolic flexibility of immune cells and how this, in turn, can impact viral replication. The possibility of a link between immunometabolism and control of viral replication is supported by research in the HIV field. Upregulation of genes associated with activation, exhaustion, and glycolysis in cells has been identified in non-controllers, individuals that fail to suppress viral loads. While HIV-specific $CD8^+$ T cells from non-controllers are predominantly glucose dependent, HIV-specific $CD8^+$ T cells from controllers have a wider diversity of metabolic resources, or metabolic flexibility, that increases their survival potential and capacity to mount responses that control HIV. Reports in the literature indicate that proteins encoded by SARS-CoV localize to the host cell mitochondria and alter mitochondrial function, limiting host cell interferon responses, and thus helping the virus evade host innate immunity.[7]

The progression of disease severity in patients with COVID-19 is frequently associated with an excessive inflammatory response and hyperactivation of monocyte-derived macrophages, with high circulating levels of inflammatory markers including C-reactive protein, ferritin, and D-dimers, inflammatory cytokines, and chemokines.[8] One could speculate that in obesity, excess nutrients like fat and carbohydrates prime immune cells to promptly initiate inflammatory cytokine and chemokine cascades. For instance, inflammasomes, large multiprotein complexes that regulate the activation of caspase-1 and enhance the proteolytic cleavage and the secretion of IL-1β and IL-18 thereby promoting immune cell infiltration and maintenance of an inflammatory microenvironment, are potential targets for nutritional modification.[9] Though essential in coordination of innate host response to infection, dysregulation of inflammasome activation can lead to defective (excessive or protracted) immune responses. Recent studies indicate that the inflammasomes can be activated by fatty acids and high glucose levels linking metabolic danger signals to the activation of inflammation and cancer development.[9] In this model, one could propose that dietary modulation of inflammasome activation contributes to pathophysiology of disease, and consequently may predispose the host to an exaggerated or abnormal response to subsequent immune challenges such as COVID.

This is just one aspect of disease process that physiologists will play a critical role in unraveling — and this is a perfect opportunity for cross collaborations between physiologists, epidemiologists, virologists, and nutritionists, to name a few. Ultimately, it is the FUNCTION of multiple cells (i.e., immune, epithelial, endothelial, etc.) that dictates progression of pathophysiological mechanisms underlying organ failure in the SARS2-infected host.

**Acknowledgments**

The author gratefully acknowledges editorial assistance from Rebecca Gonzales.

**Funding**

The author acknowledges funding from the National Institute on Alcohol Abuse and Alcoholism (NIAAA) of the National Institutes of Health (NIH) under award number P60 AA009803, “LSUHSC-NO Comprehensive Alcohol-HIV/AIDS Research Center.

**Conflicts of Interest**

None declared.

**Figure 1.**

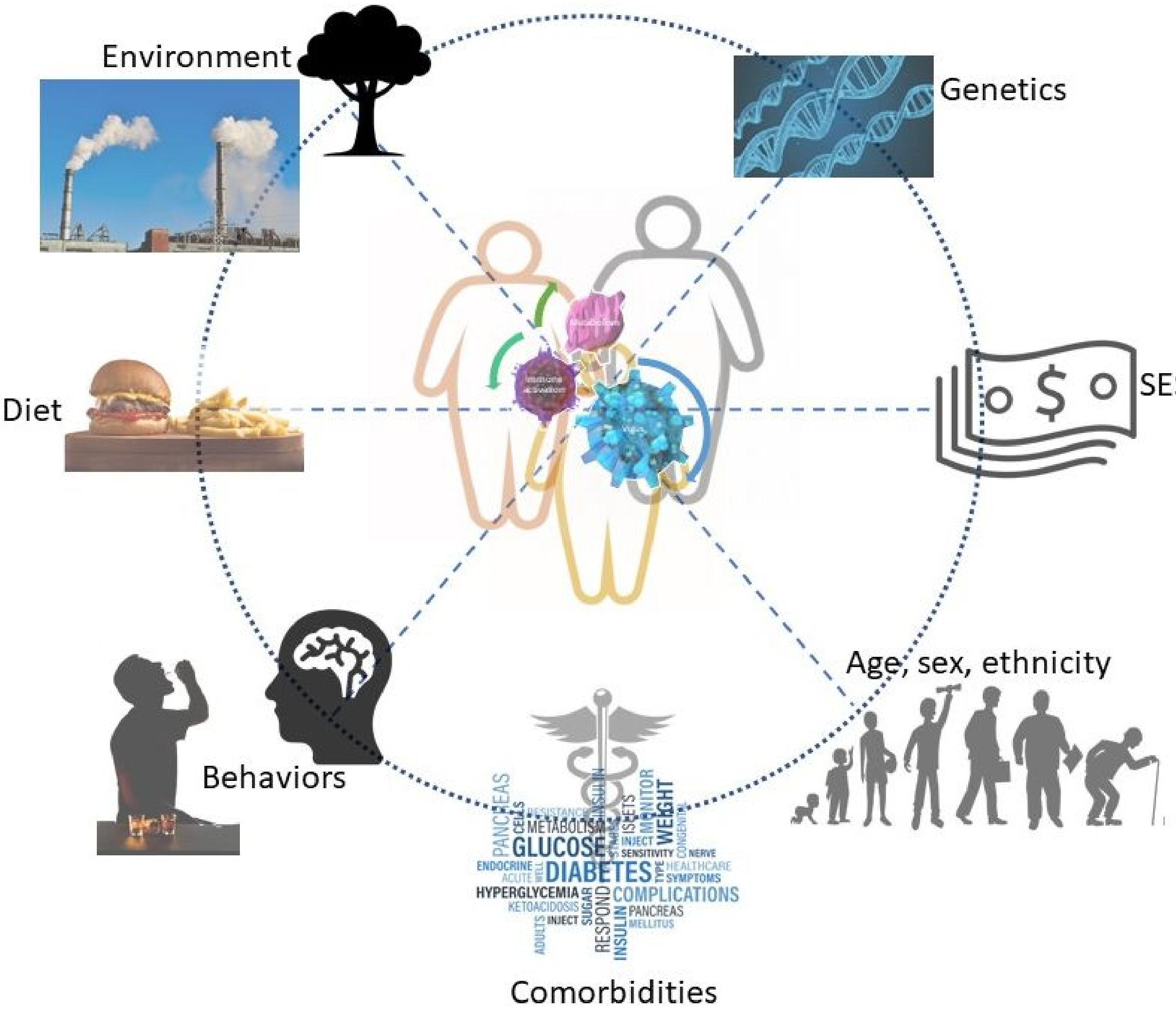

**Figure 1 Legend.**

Morbidity and mortality from SARS-CoV-2 are of greater severity in persons of color (i.e., African Americans and Hispanics). Structural and biological factors as well as behavioral and medical conditions contribute to risk factors for infection and influence the host response during infection. At the core of sustaining enhanced immune responses to viral infection and tissue injury, disrupted cellular energetics are poised to be a common functional pathophysiological mechanism contributing to severity of disease. SES; socioeconomic status.

“Money” by David, “Caduceus” by Mahmure Alp, “Brain” by Creative Stall, “Tree” by ohyeahicon from the Noun Project are licensed under CC BY-ND 2.0.

### References

1. Williamson EJ, Walker AJ, Bhaskaran K, et al. Factors associated with COVID-19-related death using OpenSAFELY. *Nature.* 2020;584 (7821):430-436. doi: 10.1038/s41586-020-2521-4.
2. Centers for Disease Control and Prevention. Coronavirus disease 2019 (COVID-19): People with certain medical conditions. https://www.cdc.gov/coronavirus/2019-ncov/need-extra-precautions/people-with-medical-conditions.html. Published 2020. Updated August 14, 2020. Accessed August 20, 2020.
3. Newsholme P, Curi R, Gordon S, Newsholme EA. Metabolism of glucose, glutamine, long-chain fatty acids and ketone bodies by murine macrophages. *Biochem J.* 1986;239 (1):121-125. doi: 10.1042/bj2390121.
4. Domblides C, Lartigue L, Faustin B. Metabolic stress in the immune function of T cells, macrophages and dendritic cells. *Cells.* 2018;7 (7). doi: 10.3390/cells7070068.
5. Liu Y, Yan LM, Wan L, et al. Viral dynamics in mild and severe cases of COVID-19. *Lancet Infect Dis.* 2020;20 (6):656-657. doi: 10.1016/s1473-3099(20)30232-2.
6. Tiku V, Tan MW, Dikic I. Mitochondrial functions in infection and immunity. *Trends Cell Biol.* 2020;30 (4):263-275. doi: 10.1016/j.tcb.2020.01.006.
7. Shi CS, Qi HY, Boularan C, et al. SARS-coronavirus open reading frame-9b suppresses innate immunity by targeting mitochondria and the MAVS/TRAF3/TRAF6 signalosome. *J Immunol.* 2014;193 (6):3080-3089. doi: 10.4049/jimmunol.1303196.
8. Merad M, Martin JC. Pathological inflammation in patients with COVID-19: A key role for monocytes and macrophages. *Nat Rev Immunol.* 2020;20 (6):355-362. doi: 10.1038/s41577-020-0331-4.
9. Christgen S, Place DE, Kanneganti TD. Toward targeting inflammasomes: Insights into their regulation and activation. *Cell Res.* 2020;30 (4):315-327. doi: 10.1038/s41422-020-0295-8.
